# Supplementary material for: Appropriateness of Prescribing Transmucosal Immediate-Release Fentanyl in the Emergency Room, During Hospitalization, and at Discharge: A Retrospective Study
Source: Pharmaceuticals (Basel). 2024 Nov 28;17(12):1609. doi: 10.3390/ph17121609 (PMC11677940; doi:10.3390/ph17121609)
Supplement: Supplementary file 1 [file pharmaceuticals-17-01609-s001.zip › pharmaceuticals-3304532-supplementary.pdf]

Supplementary Materials:

Table S1: Analysis by age range.

Variable	18-44	45-64	65-79	80+	P-Value
**Number of patients**	5	42	55	38	
**Number of episodes (%)**					0,217
**1**	5 (100.0%)	31 (73.8%)	51 (92.7%)	33 (86.8%)	
**2**	0 (0.0%)	6 (14.3%)	3 (5.5%)	3 (7.9%)	
**3**	0 (0.0%)	2 (4.8%)	1 (1.8%)	2 (5.3%)	
**5**	0 (0.0%)	3 (7.1%)	0 (0.0%)	0 (0.0%)	
**Year 2022 (%)**	2 (40.0%)	18 (42.9%)	38 (69.1%)	12 (31.6%)	0.003
**Month (%)**					0.160
**January**	1 (20.0%)	1 (2.4%)	5 (9.1%)	5 (13.2%)	
**February**	1 (20.0%)	2 (4.8%)	10 (18.2%)	2 (5.3%)	
**March**	0 (0.0%)	3 (7.1%)	2 (3.6%)	3 (7.9%)	
**April**	1 (20.0%)	6 (14.3%)	3 (5.5%)	5 (13.2%)	
**May**	0 (0.0%)	2 (4.8%)	7 (12.7%)	4 (10.5%)	
**June**	0 (0.0%)	1 (2.4%)	1 (1.8%)	2 (5.3%)	
**July**	0 (0.0%)	8 (19.0%)	2 (3.6%)	2 (5.3%)	
**August**	0 (0.0%)	4 (9.5%)	3 (5.5%)	2 (5.3%)	
**September**	2 (40.0%)	4 (9.5%)	1 (1.8%)	4 (10.5%)	
**October**	0 (0.0%)	5 (11.9%)	6 (10.9%)	3 (7.9%)	
**November**	0 (0.0%)	3 (7.1%)	11 (20.0%)	3 (7.9%)	
**December**	0 (0.0%)	3 (7.1%)	4 (7.3%)	3 (7.9%)	
**ER TIRF MME (mean (SD))**	3.38 (1.74)	7.67 (7.77)	5.89 (3.32)	6.81 (4.78)	0.220
**ER TIRF DDD (mean (SD))**	0.43 (0.22)	0.98 (1.00)	0.75 (0.43)	0.87 (0.61)	0.220
**Average Prescribing Time in Days (mean (SD))**	1.20 (2.68)	302.56 (692.85)	82.59 (135.12)	82.92 (212.76)	0.036
**Prior TIRF MME (mean (SD))**	3.12 (6.98)	5.25 (6.81)	3.24 (3.28)	2.66 (4.31)	0.104
**Prior TIRF DDD(mean (SD))**	0.40 (0.89)	0.67 (0.87)	0.42 (0.42)	0.34 (0.55)	0.104
**Prior Overall MME (mean (SD))**	76.32 (69.89)	83.33 (134.40)	61.13 (71.00)	62.10 (80.67)	0.682
**MME TIRF in Hospitalization (mean (SD))**	3.64 (6.78)	5.65 (6.72)	4.31 (5.79)	4.74 (4.29)	0.680
**Hospitalization TIRF DDD(mean (SD))**	0.47 (0.87)	0.72 (0.86)	0.53 (0.74)	0.61 (0.55)	0.601
**Discharge TIRF MME(mean (SD))**	0.00 (0.00)	4.34 (6.09)	4.23 (4.33)	1.92 (3.42)	0.022

**DDD TIRF at Discharge (mean (SD))**	0.00 (0.00)	0.56 (0.78)	0.54 (0.55)	0.25 (0.44)	0.022
**Discharge Overall MME(mean (SD))**	57.50 (84.30)	96.66 (108.45)	65.24 (70.31)	65.76 (82.00)	0.273
**Adverse Effects (%)**	0 (0.0%)	1 (2.4%)	3 (5.5%)	3 (7.9%)	0.668
**TIRF Changes (%)**					0.176
**Increase**	0 (0.0%)	4 (9.5%)	5 (9.1%)	1 (2.6%)	
**Deprescription**	0 (0.0%)	6 (14.3%)	5 (9.1%)	0 (0.0%)	
**Dies**	1 (20.0%)	4 (9.5%)	6 (10.9%)	9 (23.7%)	
**Prescription**	0 (0.0%)	3 (7.1%)	13 (23.6%)	4 (10.5%)	
**Reduction**	0 (0.0%)	3 (7.1%)	2 (3.6%)	2 (5.3%)	
**No changes**	4 (80.0%)	22 (52.4%)	24 (43.6%)	22 (57.9%)	
**NSAID intolerance (%)**	0 (0.0%)	2 (4.8%)	4 (7.3%)	5 (13.2%)	0.478
**Opioid intolerance (%)**	0 (0.0%)	1 (2.4%)	1 (1.8%)	5 (13.2%)	0.061
**Hospitalization ward (%)**					<0.001
**No hospitalization**	0 (0.0%)	15 (35.7%)	23 (41.8%)	9 (23.7%)	
**General and Digestive Surgery**	1 (20.0%)	2 (4.8%)	0 (0.0%)	1 (2.6%)	
**Vascular Surgery**	0 (0.0%)	0 (0.0%)	3 (5.5%)	0 (0.0%)	
**Palliative care**	1 (20.0%)	3 (7.1%)	8 (14.5%)	9 (23.7%)	
**Geriatrics**	0 (0.0%)	0 (0.0%)	0 (0.0%)	6 (15.8%)	
**Haematology**	0 (0.0%)	1 (2.4%)	2 (3.6%)	0 (0.0%)	
**Internal Medicine**	0 (0.0%)	1 (2.4%)	3 (5.5%)	4 (10.5%)	
**Nephrology**	0 (0.0%)	2 (4.8%)	0 (0.0%)	0 (0.0%)	
**Neurosurgery**	1 (20.0%)	0 (0.0%)	0 (0.0%)	0 (0.0%)	
**Oncology**	2 (40.0%)	15 (35.7%)	14 (25.5%)	4 (10.5%)	
**Radiation Oncology**	0 (0.0%)	1 (2.4%)	1 (1.8%)	0 (0.0%)	
**Psychiatry**	0 (0.0%)	1 (2.4%)	0 (0.0%)	0 (0.0%)	
**Traumatology**	0 (0.0%)	0 (0.0%)	0 (0.0%)	3 (7.9%)	
**Urology**	0 (0.0%)	1 (2.4%)	1 (1.8%)	2 (5.3%)	
**Age (mean (SD))**	39.00 (3.94)	55.00 (6.16)	72.33 (4.41)	85.55 (4.25)	<0.001
**Cause of pain (%)**					0.120
**Car Accident**	0 (0.0%)	1 (2.4%)	0 (0.0%)	0 (0.0%)	
**Adrenal crisis**	0 (0.0%)	1 (2.4%)	0 (0.0%)	0 (0.0%)	
**Diverticulitis**	0 (0.0%)	1 (2.4%)	0 (0.0%)	0 (0.0%)	
**Post-surgical spinal cord pain**	0 (0.0%)	0 (0.0%)	0 (0.0%)	1 (2.6%)	
**Pain secondary to treatment**	0 (0.0%)	1 (2.4%)	0 (0.0%)	1 (2.6%)	
**COPD**	0 (0.0%)	0 (0.0%)	0 (0.0%)	1 (2.6%)	
**Fracture**	0 (0.0%)	0 (0.0%)	1 (1.8%)	1 (2.6%)	
**Heart failure**	0 (0.0%)	0 (0.0%)	0 (0.0%)	3 (7.9%)	
**Lumbago**	1 (20.0%)	4 (9.5%)	3 (5.5%)	9 (23.7%)	

**Obstruction**	0 (0.0%)	0 (0.0%)	0 (0.0%)	1 (2.6%)	
**Oligoarthritis**	0 (0.0%)	1 (2.4%)	0 (0.0%)	0 (0.0%)	
**Oncological**	4 (80.0%)	29 (69.0%)	47 (85.5%)	19 (50.0%)	
**Chronic pancreatitis**	0 (0.0%)	3 (7.1%)	0 (0.0%)	0 (0.0%)	
**Colon perforation**	0 (0.0%)	0 (0.0%)	0 (0.0%)	1 (2.6%)	
**PET/Polyneuropathy**	0 (0.0%)	1 (2.4%)	0 (0.0%)	0 (0.0%)	
**Uropathy**	0 (0.0%)	0 (0.0%)	0 (0.0%)	1 (2.6%)	
**Vascular**	0 (0.0%)	0 (0.0%)	4 (7.3%)	0 (0.0%)	
**Male (%)**	4 (80.0%)	18 (42.9%)	38 (69.1%)	15 (39.5%)	0.008
**Smoking (%)**	3 (60.0%)	18 (42.9%)	19 (34.5%)	2 (5.3%)	0.001
**Mental Health (%)**	0 (0.0%)	13 (31.0%)	2 (3.6%)	4 (10.5%)	0.001
**Oncological Diagnosis (%)**	4 (80.0%)	31 (73.8%)	51 (92.7%)	23 (60.5%)	0.003
**Baseline treatment (%)**	3 (60.0%)	33 (78.6%)	47 (85.5%)	31 (81.6%)	0.506
**>60 MME**	3 (60.0%)	18 (42.9%)	20 (36.4%)	16 (42.1%)	0.726
**0 – 60 MME**	0 (0.0%)	15 (35.7%)	27 (49.1%)	15 (39.5%)	0.139
**0 MME**	2 (40.0%)	9 (21.4%)	8 (14.5%)	7 (18.4%)	0.506
**Appropriateness to indication (%)**	2 (40.0%)	12 (28.6%)	20 (36.4%)	12 (31.6%)	0.849
**Benzodiazepines (%)**	1 (20.0%)	14 (33.3%)	11 (20.0%)	14 (36.8%)	0.272
**Z drugs (%)**	0 (0.0%)	0 (0.0%)	1 (1.8%)	0 (0.0%)	0.669
**Antirheumatic (%)**	0 (0.0%)	1 (2.4%)	0 (0.0%)	0 (0.0%)	0.503
**Antidepressants (%)**	0 (0.0%)	10 (23.8%)	15 (27.3%)	6 (15.8%)	0.359
**Beta-blockers (%)**	0 (0.0%)	1 (2.4%)	4 (7.3%)	5 (13.2%)	0.273
**Oral antidiabetics (%)**	0 (0.0%)	2 (4.8%)	4 (7.3%)	1 (2.6%)	0.726
**Gabepentinoids (%)**	2 (40.0%)	8 (19.0%)	10 (18.2%)	8 (21.1%)	0.702
**CYP3A4 inhibitors (%)**	0 (0.0%)	3 (7.1%)	3 (5.5%)	5 (13.2%)	0.499

Table S2: Analysis for the presence or absence of prior (pre-hospitalization) TIRF.

**Variable**	**Prior TIRF**	**No prior TIRF**	**P-Value**
**Number of patients**	82	58	
**ER TIRF MME (mean (SD))**	7.08 (6.18)	5.87 (4.00)	0.193
**ER TIRF DDD (mean (SD))**	0.91 (0.79)	0.75 (0.51)	0.193
**Average Prescribing Time in Days (mean (SD))**	241.15 (513.80)	0.00 (0.00)	0.001
**Prior TIRF MME (mean (SD))**	6.28 (5.18)	0.00 (0.00)	<0.001
**Prior TIRF DDD(mean (SD))**	0.81 (0.66)	0.00 (0.00)	<0.001
**Prior Overall MME (mean (SD))**	96.02 (110.68)	29.81 (50.63)	<0.001

**MME TIRF in Hospitalization (mean (SD))**	5.47 (6.44)	3.88 (4.48)	0.110
**Hospitalization TIRF DDD (mean (SD))**	0.68 (0.83)	0.50 (0.57)	0.141
**Discharge TIRF MME(mean (SD))**	4.54 (5.39)	2.00 (3.25)	0.002
**Discharge TIRF DDD (mean (SD))**	0.58 (0.69)	0.26 (0.42)	0.002
**Discharge Overall MME (mean (SD))**	91.23 (98.48)	50.92 (61.46)	0.007
**Age (mean (SD))**	68.10 (12.59)	71.55 (15.57)	0.150
**Number of episodes (%)**			0.005
**1**	63 (76.8)	57 (98.3)	
**2**	11 (13.4)	1 (1.7)	
**3**	5 (6.1)	0 (0.0)	
**5**	3 (3.7)	0 (0.0)	
**Year 2022 (%)**	38 (46.3)	32 (55.2)	0.391
**Month (%)**			0.092
**January**	6 (7.3)	6 (10.3)	
**February**	12 (14.6)	3 (5.2)	
**March**	8 (9.8)	0 (0.0)	
**April**	7 (8.5)	8 (13.8)	
**May**	7 (8.5)	6 (10.3)	
**June**	3 (3.7)	1 (1.7)	
**July**	5 (6.1)	7 (12.1)	
**August**	8 (9.8)	1 (1.7)	
**September**	5 (6.1)	6 (10.3)	
**October**	8 (9.8)	6 (10.3)	
**November**	9 (11.0)	8 (13.8)	
**December**	4 (4.9)	6 (10.3)	
**Adverse effects (%)**	4 (4.9)	3 (5.2)	
**TIRF Changes (%)**			1.000
**Increase**	10 (12.2)	0 (0.0)	<0.001
**Deprescription**	11 (13.4)	0 (0.0)	
**Dies**	13 (15.9)	7 (12.1)	
**Prescription**	0 (0.0)	20 (34.5)	
**Reduction**	7 (8.5)	0 (0.0)	
**No changes**	41 (50.0)	31 (53.4)	
**NSAID intolerance (%)**	7 (8.5)	4 (6.9)	
**Opioid intolerance (%)**	4 (4.9)	3 (5.2)	0.971
**Hospitalization ward (%)**			1.000
**No hospitalization**	23 (28.0)	24 (41.4)	0.034
**General and Digestive Surgery**	2 (2.4)	2 (3.4)	
**Vascular Surgery**	2 (2.4)	1 (1.7)	
**Palliative care**	13 (15.9)	8 (13.8)	
**Geriatrics**	2 (2.4%)	4 (6.9%)	
**Haematology**	3 (3.7%)	0 (0.0%)	
**Internal Medicine**	2 (2.4%)	6 (10.3%)	
**Nephrology**	2 (2.4%)	0 (0.0%)	
**Neurosurgery**	0 (0.0%)	1 (1.7%)	
**Oncology**	28 (34.1%)	7 (12.1%)	
**Radiation Oncology**	0 (0.0%)	2 (3.4%)	
**Psychiatry**	0 (0.0%)	1 (1.7%)	
**Traumatology**	2 (2.4%)	1 (1.7%)	
**Urology**	3 (3.7%)	1 (1.7%)	

**Age Range (%)**			
**18-44**	1 (1.2%)	4 (6.9%)	0.027
**45-64**	30 (36.6%)	12 (20.7%)	
**65-79**	34 (41.5%)	21 (36.2%)	
**80+**	17 (20.7%)	21 (36.2%)	
**Cause of pain (%)**			
**Car Accident**	0 (0.0%)	1 (1.7%)	0.007
**Adrenal crisis**	1 (1.2%)	0 (0.0%)	
**Diverticulitis**	1 (1.2%)	0 (0.0%)	
**Post-surgical spinal cord pain**	1 (1.2%)	0 (0.0%)	
**Pain secondary to treatment**	2 (2.4%)	0 (0.0%)	
**COPD**	1 (1.2%)	0 (0.0%)	
**Fracture**	1 (1.2%)	1 (1.7%)	
**Heart failure**	0 (0.0%)	3 (5.2%)	
**Lumbago**	3 (3.7%)	14 (24.1%)	
**Obstruction**	0 (0.0%)	1 (1.7%)	
**Oligoarthritis**	0 (0.0%)	1 (1.7%)	
**Oncological**	66 (80.5%)	33 (56.9%)	
**Chronic pancreatitis**	3 (3.7%)	0 (0.0%)	
**Colon perforation**	0 (0.0%)	1 (1.7%)	
**PET/Polyneuropathy**	1 (1.2%)	0 (0.0%)	
**Uropathy**	0 (0.0%)	1 (1.7%)	
**Vascular**	2 (2.4%)	2 (3.4%)	
**Male (%)**	45 (54.9%)	30 (51.7%)	0.844
**Smoking (%)**	22 (26.8%)	20 (34.5%)	0.432
**Mental Health (%)**	11 (13.4%)	8 (13.8%)	1.000
**Oncological Diagnosis (%)**	72 (87.8%)	37 (63.8%)	0.002
**Baseline treatment (%)**	74 (90.2%)	40 (69.0%)	0.003
**>60 MME**	46 (56.1%)	11 (19.0%)	<0.001
**0 – 60 MME**	28 (34.1%)	29 (50.0%)	0.088
**0 MME**	8 (9.8%)	18 (31.0%)	0.003
**Appropriateness to indication (%)**	40 (48.8%)	6 (10.3%)	<0.001
**Benzodiazepines (%)**	28 (34.1%)	12 (20.7%)	0.122
**Z drugs (%)**	0 (0.0%)	1 (1.7%)	0.861
**Antirheumatic (%)**	0 (0.0%)	1 (1.7%)	0.861
**Antidepressants (%)**	25 (30.5%)	6 (10.3%)	0.009
**Beta-blockers (%)**	7 (8.5%)	3 (5.2%)	0.668
**Oral antidiabetics (%)**	4 (4.9%)	3 (5.2%)	1.000
**Gabapentinoids (%)**	17 (20.7%)	11 (19.0%)	0.966
**CYP3A4 inhibitors (%)**	8 (9.8%)	3 (5.2%)	0.500

Table S3: Patients characteristics by hospitalization status.

Variable	Without hospitalization	With hospitalization	P-Value
**Number of patients**	47	93	
**ER TIRF MME (mean (SD))**	5.31 (4.38)	7.22 (5.77)	0.048
**ER TIRF DDD (mean (SD))**	0.68 (0.56)	0.93 (0.74)	0.048
**Average Prescribing Time in Days (mean (SD))**	79.79 (190.25)	176.59 (483.42)	0.213
**Prior TIRF MME (mean (SD))**	2.38 (3.23)	4.34 (5.63)	0.029
**Prior TIRF DDD(mean (SD))**	0.30 (0.41)	0.56 (0.72)	0.029
**Prior Overall MME (mean (SD))**	57.44 (111.14)	74.23 (87.86)	0.331

**MME TIRF in Hospitalization (mean (SD))**	0.00 (0.00)	7.14 (5.69)	<0.001
**Hospitalization TIRF DDD(mean (SD))**	0.00 (0.00)	0.90 (0.73)	<0.001
**Discharge TIRF MME(mean (SD))**	3.78 (3.65)	3.34 (5.27)	0.608
**Discharge TIRF DDD (mean (SD))**	0.48 (0.47)	0.43 (0.68)	0.608
**Discharge Overall MME (mean (SD))**	66.07 (82.93)	78.81 (89.36)	0.416
**Age (mean (SD))**	69.40 (12.72)	69.59 (14.60)	0.941
**Number of episodes (%)**			0.512
**1**	41 (87.2)	79 (84.9)	
**2**	5 (10.6)	7 (7.5)	
**3**	1 (2.1)	4 (4.3)	
**5**	0 (0.0)	3 (3.2)	
**Year 2022 (%)**	29 (61.7)	41 (44.1)	0.074
**Month (%)**			0.687
**January**	5 (10.6)	7 (7.5)	
**February**	6 (12.8)	9 (9.7)	
**March**	3 (6.4)	5 (5.4)	
**April**	5 (10.6)	10 (10.8)	
**May**	3 (6.4)	10 (10.8)	
**June**	0 (0.0)	4 (4.3)	
**July**	7 (14.9)	5 (5.4)	
**August**	3 (6.4)	6 (6.5)	
**September**	2 (4.3)	9 (9.7)	
**October**	4 (8.5)	10 (10.8)	
**November**	5 (10.6)	12 (12.9)	
**December**	4 (8.5)	6 (6.5)	
**Adverse effects (%)**	1 (2.1)	6 (6.5)	0.485
**TIRF Changes (%)**			0.004
**Increase**	2 (4.3)	8 (8.6)	
**Deprescription**	2 (4.3)	9 (9.7)	
**Dies**	3 (6.4)	17 (18.3)	
**Prescription**	14 (29.8)	6 (6.5)	
**Reduction**	3 (6.4)	4 (4.3)	
**No changes**	23 (48.9)	49 (52.7)	
**NSAID intolerance (%)**	8 (17.0)	3 (3.2)	0.011
**Opioid intolerance (%)**	5 (10.6)	2 (2.2)	0.077
**Hospitalization ward (%)**			<0.001
**No hospitalization**	47 (100.0)	0 (0.0)	
**General and Digestive Surgery**	0 (0.0)	4 (4.3)	
**Vascular Surgery**	0 (0.0)	3 (3.2)	
**Palliative care**	0 (0.0)	21 (22.6)	
**Geriatrics**	0 (0.0)	6 (6.5)	
**Haematology**	0 (0.0)	3 (3.2)	
**Internal Medicine**	0 (0.0)	8 (8.6)	
**Nephrology**	0 (0.0)	2 (2.2)	
**Neurosurgery**	0 (0.0)	1 (1.1)	
**Oncology**	0 (0.0)	35 (37.6)	
**Radiation Oncology**	0 (0.0)	2 (2.2)	
**Psychiatry**	0 (0.0)	1 (1.1)	
**Traumatology**	0 (0.0)	3 (3.2)	
**Urology**	0 (0.0)	4 (4.3)	

**Age Range (%)**			0.114
**18-44**	0 (0.0)	5 (5.4)	
**45-64**	15 (31.9)	27 (29.0)	
**65-79**	23 (48.9)	32 (34.4)	
**80+**	9 (19.1)	29 (31.2)	
**Cause of pain (%)**			0.547
**Car Accident**	1 (2.1)	0 (0.0)	
**Adrenal crisis**	1 (2.1)	0 (0.0)	
**Diverticulitis**	0 (0.0)	1 (1.1)	
**Post-surgical spinal cord pain**	0 (0.0)	1 (1.1)	
**Pain secondary to treatment**	1 (2.1)	1 (1.1)	
**COPD**	0 (0.0)	1 (1.1)	
**Fracture**	1 (2.1)	1 (1.1)	
**Heart failure**	1 (2.1)	2 (2.2)	
**Lumbago**	8 (17.0)	9 (9.7)	
**Obstruction**	0 (0.0)	1 (1.1)	
**Oligoarthritis**	1 (2.1)	0 (0.0)	
**Oncological**	31 (66.0)	68 (73.1)	
**Chronic pancreatitis**	0 (0.0)	3 (3.2)	
**Colon perforation**	1 (2.1)	0 (0.0)	
**PET/Polyneuropathy**	0 (0.0)	1 (1.1)	
**Uropathy**	0 (0.0)	1 (1.1)	
**Vascular**	1 (2.1)	3 (3.2)	
**Male (%)**	23 (48.9)	52 (55.9)	0.547
**Smoking (%)**	17 (36.2)	25 (26.9)	0.349
**Mental Health (%)**	7 (14.9)	12 (12.9)	0.949
**Oncological Diagnosis (%)**	33 (70.2)	76 (81.7)	0.182
**Baseline treatment (%)**	37 (78.7)	77 (82.8)	0.723
**>60 MME**	13 (27.7)	44 (47.3)	0.040
**0 – 60 MME**	24 (51.1)	33 (35.5)	0.112
**0 MME**	10 (21.3)	16 (17.2)	0.723
**Appropriateness to indication (%)**	11 (23.4)	35 (37.6)	0.133
**Benzodiazepines (%)**	9 (19.1)	31 (33.3)	0.120
**Z drugs (%)**	0 (0.0)	1 (1.1)	1.000
**Antirheumatic (%)**	1 (2.1)	0 (0.0)	0.727
**Antidepressants (%)**	11 (23.4)	20 (21.5)	0.968
**Beta-blockers (%)**	3 (6.4)	7 (7.5)	1.000
**Oral antidiabetics (%)**	4 (8.5)	3 (3.2)	0.345
**Gabepentinoids (%)**	8 (17.0)	20 (21.5)	0.687
**CYP3A4 inhibitors (%)**	3 (6.4)	8 (8.6)	0.898

Table S4: Analysis for changes in TIRF at Discharge.

Variable	Increase	Deprescription	Deaths	Prescription	Reduction	No changes	P-Value
**Number of patients**	10	11	20	20	7	72	
**Number of episodes (%)**							0.761
**1**	8 (80.0)	9 (81.8)	17 (85.0)	19 (95.0)	6 (85.7)	61 (84.7)	

**2**	2 (20.0)	2 (18.2)	2 (10.0)	1 (5.0)	0 (0.0)	5 (6.9)	
**3**	0 (0.0)	0 (0.0)	1 (5.0)	0 (0.0)	1 (14.3)	3 (4.2)	
**5**	0 (0.0)	0 (0.0)	0 (0.0)	0 (0.0)	0 (0.0)	3 (4.2)	
**Year 2022 (%)**	6 (60.0)	5 (45.5)	7 (35.0)	13 (65.0)	3 (42.9)	36 (50.0)	0.516
**Month (%)**							0.447
**January**	0 (0.0)	2 (18.2)	3 (15.0)	1 (5.0)	1 (14.3)	5 (6.9)	
**February**	3 (30.0)	1 (9.1)	2 (10.0)	3 (15.0)	0 (0.0)	6 (8.3)	
**March**	0 (0.0)	1 (9.1)	1 (5.0)	0 (0.0)	2 (28.6)	4 (5.6)	
**April**	1 (10.0)	0 (0.0)	2 (10.0)	1 (5.0)	1 (14.3)	10 (13.9)	
**May**	1 (10.0)	1 (9.1)	1 (5.0)	3 (15.0)	0 (0.0)	7 (9.7)	
**June**	0 (0.0)	0 (0.0)	3 (15.0)	0 (0.0)	0 (0.0)	1 (1.4)	
**July**	1 (10.0)	1 (9.1)	0 (0.0)	4 (20.0)	0 (0.0)	6 (8.3)	
**August**	1 (10.0)	1 (9.1)	1 (5.0)	0 (0.0)	0 (0.0)	6 (8.3)	
**September**	0 (0.0)	1 (9.1)	1 (5.0)	0 (0.0)	2 (28.6)	7 (9.7)	
**October**	0 (0.0)	2 (18.2)	2 (10.0)	3 (15.0)	0 (0.0)	7 (9.7)	
**November**	1 (10.0)	1 (9.1)	3 (15.0)	3 (15.0)	1 (14.3)	8 (11.1)	
**December**	2 (20.0)	0 (0.0)	1 (5.0)	2 (10.0)	0 (0.0)	5 (6.9)	
**ER TIRF MME (mean (SD))**	6.11 (3.93)	8.86 (5.42)	7.54 (4.33)	5.14 (2.84)	7.80 (10.72)	6.31 (5.68)	0.467
**ER TIRF DDD (mean (SD))**	0.78 (0.50)	1.14 (0.69)	0.97 (0.56)	0.66 (0.36)	1.00 (1.37)	0.81 (0.73)	0.467
**Average Prescribing Time in Days (mean (SD))**	194.60 (233.06)	178.36 (224.92)	57.80 (100.74)	0.00 (0.00)	217.43 (219.84)	183.17 (545.56)	0.597
**Prior TIRF MME (mean (SD))**	3.32 (1.73)	6.26 (2.84)	4.75 (5.05)	0.00 (0.00)	7.24 (10.70)	3.71 (5.01)	0.002
**Prior TIRF DDD(mean (SD))**	0.43 (0.22)	0.80 (0.36)	0.61 (0.65)	0.00 (0.00)	0.93 (1.37)	0.48 (0.64)	0.002
**Prior Overall MME (mean (SD))**	102.82 (218.82)	58.86 (57.08)	56.52 (67.04)	18.14 (19.19)	113.14 (154.45)	78.36 (83.99)	0.09
**MME TIRF in Hospitalization (mean (SD))**	6.50 (9.01)	6.62 (6.08)	7.59 (5.26)	3.15 (5.31)	2.97 (5.90)	4.18 (5.16)	0.085
**Hospitalization TIRF DDD(mean (SD))**	0.83 (1.15)	0.85 (0.78)	0.90 (0.70)	0.40 (0.68)	0.38 (0.76)	0.54 (0.66)	0.155
**Discharge TIRF MME(mean (SD))**	8.39 (5.74)	0.00 (0.00)	0.00 (0.00)	5.79 (2.94)	2.97 (5.90)	3.71 (5.01)	<0.001
**Discharge TIRF DDD (mean (SD))**	1.08 (0.74)	0.00 (0.00)	0.00 (0.00)	0.74 (0.38)	0.38 (0.76)	0.48 (0.64)	<0.001
**Discharge Overall MME(mean (SD))**	119.15 (147.61)	89.44 (54.45)	0.00 (0.00)	55.79 (44.64)	101.19 (145.26)	89.37 (84.30)	<0.001
**Adverse effects (%)**	1 (10.0)	0 (0.0)	1 (5.0)	1 (5.0)	0 (0.0)	4 (5.6)	0.911

**TIRF Changes (%)**							<0.001
**Increase**	10 (100.0)	0 (0.0)	0 (0.0)	0 (0.0)	0 (0.0)	0 (0.0)	
**Deprescription**	0 (0.0)	11 (100.0)	0 (0.0)	0 (0.0)	0 (0.0)	0 (0.0)	
**Dies**	0 (0.0)	0 (0.0)	20 (100.0)	0 (0.0)	0 (0.0)	0 (0.0)	
**Prescription**	0 (0.0)	0 (0.0)	0 (0.0)	20 (100.0)	0 (0.0)	0 (0.0)	
**Reduction**	0 (0.0)	0 (0.0)	0 (0.0)	0 (0.0)	7 (100.0)	0 (0.0)	
**No changes**	0 (0.0)	0 (0.0)	0 (0.0)	0 (0.0)	0 (0.0)	72 (100.0)	
**NSAID intolerance (%)**	0 (0.0)	0 (0.0)	2 (10.0)	3 (15.0)	2 (28.6)	4 (5.6)	0.156
**Opioid intolerance (%)**	0 (0.0)	0 (0.0)	1 (5.0)	0 (0.0)	2 (28.6)	4 (5.6)	0.065
**Hospitalization ward (%)**							0.208
**No hospitalization**	2 (20.0)	2 (18.2)	3 (15.0)	14 (70.0)	3 (42.9)	23 (31.9)	
**General and Digestive Surgery**	0 (0.0)	1 (9.1)	0 (0.0)	0 (0.0)	0 (0.0)	3 (4.2)	
**Vascular Surgery**	0 (0.0)	1 (9.1)	0 (0.0)	0 (0.0)	0 (0.0)	2 (2.8)	
**Palliative care**	3 (30.0)	2 (18.2)	7 (35.0)	2 (10.0)	3 (42.9)	4 (5.6)	
**Geriatrics**	0 (0.0)	0 (0.0)	0 (0.0)	0 (0.0)	0 (0.0)	6 (8.3)	
**Haematology**	0 (0.0)	0 (0.0)	0 (0.0)	0 (0.0)	0 (0.0)	3 (4.2)	
**Internal Medicine**	0 (0.0)	0 (0.0)	0 (0.0)	2 (10.0)	0 (0.0)	6 (8.3)	
**Nephrology**	0 (0.0)	0 (0.0)	0 (0.0)	0 (0.0)	0 (0.0)	2 (2.8)	
**Neurosurgery**	0 (0.0)	0 (0.0)	0 (0.0)	0 (0.0)	0 (0.0)	1 (1.4)	
**Oncology**	5 (50.0)	5 (45.5)	8 (40.0)	1 (5.0)	1 (14.3)	15 (20.8)	
**Radiation Oncology**	0 (0.0)	0 (0.0)	0 (0.0)	1 (5.0)	0 (0.0)	1 (1.4)	
**Psychiatry**	0 (0.0)	0 (0.0)	0 (0.0)	0 (0.0)	0 (0.0)	1 (1.4)	
**Traumatology**	0 (0.0)	0 (0.0)	2 (10.0)	0 (0.0)	0 (0.0)	1 (1.4)	
**Urology**	0 (0.0)	0 (0.0)	0 (0.0)	0 (0.0)	0 (0.0)	4 (5.6)	
**Age (mean (SD))**	64.90 (11.68)	62.45 (8.50)	73.15 (12.43)	74.60 (10.40)	69.57 (18.26)	68.83 (15.26)	0.149
**Age Range (%)**							0.176
**18-44**	0 (0.0)	0 (0.0)	1 (5.0)	0 (0.0)	0 (0.0)	4 (5.6)	
**45-64**	4 (40.0)	6 (54.5)	4 (20.0)	3 (15.0)	3 (42.9)	22 (30.6)	
**65-79**	5 (50.0)	5 (45.5)	6 (30.0)	13 (65.0)	2 (28.6)	24 (33.3)	
**80+**	1 (10.0)	0 (0.0)	9 (45.0)	4 (20.0)	2 (28.6)	22 (30.6)	
**Cause of pain (%)**							0.865

**Car Accident**	0 (0.0)	0 (0.0)	0 (0.0)	0 (0.0)	0 (0.0)	1 (1.4)	
**Adrenal crisis**	0 (0.0)	0 (0.0)	0 (0.0)	0 (0.0)	1 (14.3)	0 (0.0)	
**Diverticulitis**	0 (0.0)	1 (9.1)	0 (0.0)	0 (0.0)	0 (0.0)	0 (0.0)	
**Post-surgical spinal cord pain**	0 (0.0)	0 (0.0)	0 (0.0)	0 (0.0)	0 (0.0)	1 (1.4)	
**Pain secondary to treatment**	0 (0.0)	0 (0.0)	0 (0.0)	0 (0.0)	0 (0.0)	2 (2.8)	
**COPD**	0 (0.0)	0 (0.0)	0 (0.0)	0 (0.0)	0 (0.0)	1 (1.4)	
**Fracture**	0 (0.0)	0 (0.0)	1 (5.0)	1 (5.0)	0 (0.0)	0 (0.0)	
**Heart failure**	0 (0.0)	0 (0.0)	1 (5.0)	0 (0.0)	0 (0.0)	2 (2.8)	
**Lumbago**	1 (10.0)	0 (0.0)	0 (0.0)	4 (20.0)	1 (14.3)	11 (15.3)	
**Obstruction**	0 (0.0)	0 (0.0)	0 (0.0)	0 (0.0)	0 (0.0)	1 (1.4)	
**Oligoarthritis**	0 (0.0)	0 (0.0)	0 (0.0)	0 (0.0)	0 (0.0)	1 (1.4)	
**Oncological**	9 (90.0)	9 (81.8)	17 (85.0)	15 (75.0)	5 (71.4)	44 (61.1)	
**Chronic pancreatitis**	0 (0.0)	0 (0.0)	0 (0.0)	0 (0.0)	0 (0.0)	3 (4.2)	
**Colon perforation**	0 (0.0)	0 (0.0)	1 (5.0)	0 (0.0)	0 (0.0)	0 (0.0)	
**PET/Polyneuropathy**	0 (0.0)	0 (0.0)	0 (0.0)	0 (0.0)	0 (0.0)	1 (1.4)	
**Uropathy**	0 (0.0)	0 (0.0)	0 (0.0)	0 (0.0)	0 (0.0)	1 (1.4)	
**Vascular**	0 (0.0)	1 (9.1)	0 (0.0)	0 (0.0)	0 (0.0)	3 (4.2)	
**Male (%)**	6 (60.0)	5 (45.5)	12 (60.0)	12 (60.0)	2 (28.6)	38 (52.8)	0.715
**Smoking (%)**	4 (40.0)	2 (18.2)	5 (25.0)	7 (35.0)	2 (28.6)	22 (30.6)	0.889
**Mental Health (%)**	1 (10.0)	1 (9.1)	3 (15.0)	2 (10.0)	2 (28.6)	10 (13.9)	0.863
**Oncological Diagnosis (%)**	9 (90.0)	11 (100.0)	17 (85.0)	16 (80.0)	6 (85.7)	50 (69.4)	0.166
**Baseline treatment (%)**	8 (80.0)	9 (81.8)	14 (70.0)	19 (95.0)	7 (100.0)	57 (79.2)	0.304
**>60 MME**	3 (30.0)	4 (36.4)	9 (45.0)	3 (15.0)	4 (57.1)	34 (47.2)	0.144
**0 – 60 MME**	5 (50.0)	5 (45.5)	5 (25.0)	16 (80.0)	3 (42.9)	23 (31.9)	0.003
**0 MME**	2 (20.0)	2 (18.2)	6 (30.0)	1 (5.0)	0 (0.0)	15 (20.8)	0.304
**Appropriateness to indication (%)**	2 (20.0)	4 (36.4)	9 (45.0)	1 (5.0)	4 (57.1)	26 (36.1)	0.044
**Benzodiazepines (%)**	3 (30.0)	2 (18.2)	5 (25.0)	4 (20.0)	3 (42.9)	23 (31.9)	0.771
**Z drugs (%)**	0 (0.0)	0 (0.0)	0 (0.0)	0 (0.0)	0 (0.0)	1 (1.4)	0.966
**Antirheumatic (%)**	0 (0.0)	0 (0.0)	0 (0.0)	0 (0.0)	0 (0.0)	1 (1.4)	0.966
**Antidepressants (%)**	5 (50.0)	1 (9.1)	3 (15.0)	1 (5.0)	2 (28.6)	19 (26.4)	0.062
**Beta-blockers (%)**	0 (0.0)	0 (0.0)	4 (20.0)	2 (10.0)	0 (0.0)	4 (5.6)	0.176
**Oral antidiabetics (%)**	0 (0.0)	0 (0.0)	3 (15.0)	1 (5.0)	0 (0.0)	3 (4.2)	0.327

**Gabapentinoids (%)**	3 (30.0)	3 (27.3)	1 (5.0)	3 (15.0)	2 (28.6)	16 (22.2)	0.459
**CYP3A4 inhibitors (%)**	1 (10.0)	1 (9.1)	0 (0.0)	2 (10.0)	1 (14.3)	6 (8.3)	0.8
